# Effectiveness of pyronaridine-artesunate against *Plasmodium malariae, Plasmodium ovale* spp, and mixed-*Plasmodium* infections: a post-hoc analysis of the CANTAM-Pyramax trial

**DOI:** 10.1016/S2666-5247(22)00092-1

**Published:** 2022-08

**Authors:** Mirjam Groger, Gaston Tona Lutete, Ghyslain Mombo-Ngoma, Nsengi Y Ntamabyaliro, Gauthier Kahunu Mesia, Trésor Bodjick Muena Mujobu, Lia Betty Dimessa Mbadinga, Rella Zoleko Manego, Diane Egger-Adam, Isabelle Borghini-Fuhrer, Jangsik Shin, Robert Miller, Sarah Arbe-Barnes, Stephan Duparc, Michael Ramharter

**Affiliations:** a Institut für Tropenmedizin, University of Tubingen, Tubingen, Germany; b Department of Tropical Medicine, Bernhard Nocht Institute for Tropical Medicine, and Department of Internal Medicine I, University Medical Center Hamburg-Eppendorf, Hamburg, Germany; c Unité de Pharmacologie Clinique et Pharmacovigilance, University of Kinshasa, Kinshasa, Democratic Republic of the Congo; d Centre de Recherches Médicales de Lambaréné, Lambaréné, Gabon; e Medicines for Malaria Venture, Geneva, Switzerland; f Shin-Poong Pharmaceutical, Seoul, Korea; g Artemida Pharma, Stevenage, UK

## Abstract

**Background:**

High-quality evidence for the therapeutic efficacy and effectiveness of antimalarials for infections caused by *Plasmodium malariae, Plasmodium ovale* spp, and mixed-*Plasmodium* infections is scarce. In this study, we aimed to analyse the efficacy of pyronaridine–artesunate for the treatment of non-falciparum and mixed-species *Plasmodium* infections from a large phase 3b/4 clinical trial in central Africa.

**Methods:**

This post-hoc analysis was done in a random subset of samples from two sites (in the Democratic Republic of the Congo and in Gabon) of the CANTAM-Pyramax trial assessing pyronaridine-artesunate therapy. We randomly selected paired dried blood spot samples from day 0 and day 28 (or unforeseen visit) and analysed them by quantitative PCR for mixed *Plasmodium* infections or non-falciparum mono-infections. Day 28 (or unforeseen visit) samples positive for non-falciparum malaria were re-assessed by microscopy to identify microscopic versus submicroscopic infections. Analyses were done on two sample sets: a per-protocol set and an intention-to-treat set.

**Findings:**

Among 1502 randomly selected samples, 192 (12·8%) showed mixed-*Plasmodium* infections or non-falciparum mono-infections. We did not detect *P vivax* in the samples. For both the per-protocol and intention-to-treat sets, the overall day 28 cure rates for *P malariae, P ovale curtisi*, and *P ovale wallikeri* were 96·3% or higher (95% CIs from 81·0–99·9 to 95·7–100). Cure rates were consistently high in *P malariae* (99·2%, 95·7–100) and *P ovale* spp (97·9%, 88·7–99·9, for *P ovale curtisi* and 96·3%, 81·0–99·9, for *P ovale wallikeri*) infections.

**Interpretation:**

This post-hoc analysis provides important evidence supporting the high efficacy of pyronaridine-artesunate against mono-infections with *P malariae, P ovale curtisi*, or *P ovale wallikeri* and mixed-*Plasmodium* infections in a real-world setting.

**Funding:**

Medicines for Malaria Venture.

## Introduction

Over the past few decades, many clinical trials have shown the efficacy, tolerability, and safety of antimalarial therapies for the treatment of malaria caused by *Plasmodium falciparum* and, to a lesser extent, caused by *Plasmodium vivax*.^1^ Mono-infections with *Plasmodium malariae, Plasmodium ovale curtisi,* or *Plasmodium ovale wallikeri* and mixed *Plasmodium* infections have mostly been excluded from these clinical trials, and data analyses and evidence showing the efficacy of recommended treatments in these infections are thus extremely scarce.^2^, ^3^ Whereas non-falciparum *Plasmodium* species were traditionally regarded as rarely occurring species and of minor importance, the availability of diagnostic methods with increased sensitivity, changes in malaria transmission patterns, and improved control of falciparum malaria have led to an increased importance of these neglected forms of malaria.^4^, ^5^

For years, malaria cases reported from African countries were almost exclusively classified as *P falciparum*, as shown in previous WHO World Malaria Reports.^6^ This is mostly due to the difficulties in diagnosing non-falciparum malaria and mixed-species malaria. With improved diagnostic tools and gradually increasing focus on these non-falciparum species, other *Plasmodium* species are finding their way into these reports too. Over the past decade, non-falciparum and non-vivax malaria has moved in the focus of the scientific community, and publications reporting non-falciparum or mixed *Plasmodium* infections have increased, not only from falciparum-endemic countries, but also in travel returnees.^5^, ^7^, ^8^ It seems that, although the transmission of *P falciparum* is declining in Africa, the transmission of *P ovale* spp and *P malariae* persists, with an overall increase in odds of infection.^4^, ^9^

Persistence of *P ovale* spp and *P malariae* has also been reported in Asia, where asymptomatic infections have the potential to challenge progress of malaria control programmes.^10^ Although asymptomatic malaria frequently remains untreated in high-transmission settings, treatment of all *Plasmodium* infections is important to reduce morbidity and, especially in malaria-elimination settings, to interrupt transmission.^4^, ^11^

**Research in context**

**Evidence before this study**

Two previous systematic reviews have shown a scarcity of evidence for treatment recommendations for malaria caused by *Plasmodium ovale* spp and *Plasmodium malariae*. We searched WHO's International Clinical Trials Registry Platform on Oct 22, 2021, with the terms “*Plasmodium ovale*”, “*Plasmodium malariae*”, “non-falciparum”, “mixed *Plasmodium*”, and “mixed infection”, with no language restrictions, which resulted in the identification of two small prospective trials for the treatment of acute malaria with artemether–lumefantrine. With the same search terms and on the same date, we also searched the European Medicines Agency webpage and the US Food and Drug Administration webpage (using the tool FDALabel) for registered drugs, and we found no artemisinin-based combination therapies registered for the treatment of *P ovale* spp, *P malariae*, or mixed *Plasmodium* malaria.

**Added value of this study**

Over the past decade, non-falciparum and mixed *Plasmodium* infections have been reported with increasing frequency in malaria endemic countries, as well as in travellers from non-endemic countries. At the same time, evidence-based efficacious antimalarial treatments, which can be widely applied, are needed. However, antimalarial treatments for all *Plasmodium* species and mixed *Plasmodium* infections are indispensable for malaria control and elimination. We did a post-hoc analysis focused on non-falciparum and mixed *Plasmodium* infections on a large subset of participants who took part in a large, prospective, clinical phase 3b/4, cohort event monitoring study designed to evaluate, in a real-life setting, the safety, tolerability, and efficacy of pyronaridine–artesunate therapy for uncomplicated malaria.

**Implications of all the available evidence**

The results of this study provide high-quality evidence that support the use of pyronaridine–artesunate for the treatment of all African *Plasmodium* species reported in this study. This can simplify the future disease management of patients with malaria given the challenges of routine diagnostics of non-falciparum and mixed-species malaria by rapid diagnostic tests and microscopy. The nature of the analysed data highlights not only the efficacy, but also the effectiveness of pyronaridine–artesunate in a real-world setting.

A fixed dose combination of pyronaridine and artesunate (Pyramax, Seoul, South Korea) is the only artemisinin-based combination therapy with positive assessments from stringent regulatory authorities for both the treatment of *P falciparum* and blood-stage *P vivax* malaria. For malaria control and malaria elimination programmes, efficacious antimalarial treatments for all *Plasmodium* species and mixed infection are indispensable. Generating more evidence for the treatment of infections with *P malariae* and *P ovale* spp is thus a much-needed undertaking.

For this post-hoc analysis, we analysed blood samples from the CANTAM-Pyramax real-world study, in which pyronaridine–artesunate was administered to patients with acute uncomplicated malaria, to provide a patient pool of considerable size from endemic countries with a known prevalence of *P malariae* and *P ovale* spp.^12^

We aimed to assess the proportions of non-falciparum, non-vivax monoinfections and mixed *Plasmodium* infections and corresponding efficacy of pyronaridine–artesunate for infections with *Plasmodium* species and mixed infections to provide urgently needed evidence based on systematically collected data.

## Methods

### Participants

We did a post-hoc analysis assessing samples collected during the CANTAM-Pyramax trial (NCT03201770), which has been published in more detail elsewhere.^12^ In short, the CANTAM-Pyramax trial was an open label, non-randomised, phase 3b/4, cohort event monitoring study designed to evaluate, in a real-life setting, the safety, tolerability, and efficacy of pyronaridine–artesunate therapy administered daily over 3 consecutive days in patients with uncomplicated malaria. Patients were included on the basis of a positive rapid diagnostic test (RDT) result or malaria microscopy. The first dose was administered as directly observed treatment, and the subsequent two doses were taken unobserved by the participants. On day 7 (±1) and day 28 (±2), a trained community health worker visited the participants at home to monitor safety, assess compliance by collecting empty blisters and any leftover pills or sachets, and collect blood for malaria microscopy and molecular analyses. Participants were further encouraged to contact the community health worker in case of health concerns outside the scheduled visits. Participants could be re-treated in the CANTAM-Pyramax trial for subsequent malaria episodes. Therefore, blood spots from multiple malaria events might be available for a participant.

### Procedures

We did a retrospective real-time quantitative PCR (qPCR) analysis using dried blood spot (DBS) samples from the two CANTAM-Pyramax African sites: the Centre Hospitalier du Mont-Amba in Kinshasa (Democratic Republic of the Congo [DR Congo]), representing an urban setting in a large metropolitan region of central Africa, and the Centre de Recherches Médicales de Lambaréné (CERMEL) in Lambaréné (Gabon),^13^ representing a rural central African setting. These sites were selected as they had the highest recruitment rates in the CANTAM-Pyramax trial, represent different endemic settings (urban *vs* rural), and are both known regions of relevant prevalence of non-falciparum malaria.^7^, ^14^, ^15^ The CANTAM-Pyramax trial and the ancillary protocol covering this post-hoc analysis were approved by the responsible regulatory and ethics review bodies in DR Congo (reference CEUPC0048) and Gabon (number 041/2018/PR/SG/CNE), as applicable.

For this post-hoc analysis, a random subset of malaria episodes for whom paired samples from day 0 and day 28 (or day 0 and unforeseen visit between day 4 and day 28) were available was identified by generation of a random list by an independent statistician, with the aim of including 750 cases per study site.

### Sample analyses

We analysed DBS samples by qPCR at the Department of Biomedical Sciences, Institute of Tropical Medicine (Antwerp, Belgium), to detect and differentiate *P falciparum, P malariae, P ovale curtisi, P ovale wallikeri*, and *P vivax* with previously published methods.^16^, ^17^, ^18^, ^19^ Parasite counts by qPCR were species specific, and singleplex assays were used to individually identify *P falciparum* (*var*ATS, limit of detection 0·05 parasites per μL), *P vivax* (*Pv*-*mtCOX1* qPCR, 1 parasite per μL), *P malariae* (Mal qPCR, 5 parasites per μL), and *P ovale* spp (Ova, 5 parasites per μL). Samples positive for *P ovale* spp were further analysed for *P ovale curtisi* and *P ovale wallikeri*.^16^, ^17^, ^18^, ^19^ PCR analysis of samples from day 28 or unforeseen visits were only done in case the PCR analysis on day 0 detected the presence of at least one *Plasmodium* species other than *P falciparum*. For samples in which qPCR identified mixed *Plasmodium* or non-falciparum infection on day 28 (or unforeseen visit), the corresponding thick smear was reassessed for microscopic presence of non-falciparum malaria, to ascertain microscopic versus submicroscopic parasitaemia and to confirm asexual parasitaemia as opposed to assay positivity triggered by gametocytaemia alone. Microscopic reassessment was done at the Laboratoire Clinique at CERMEL, by use of the Lambaréné method for malaria microscopy.^20^

### Statistical analysis

Statistical analyses were descriptive and done with SAS (version 9.4) in a Linux environment by Datamap (Freiburg, Germany). Two analysis sets were defined for this post-hoc analysis. The post-hoc per-protocol set comprised of all malaria episodes excluding those with major protocol deviations, whereas the post-hoc intention-to-treat analysis set included all malaria episodes of the random sample subset. Clinical patient data were extracted from the CANTAM-Pyramax clinical database and provided for all malaria episodes included in the random sample subset.

Demographic data and patient characteristics for the post-hoc analysis set were summarised, with number and percentages for categorical variables, and mean (SD), median (IQR), and range for continuous variables. Results are provided for all non-falciparum and mixed-species infections overall, by study site, and by species present on day 0.

Effectiveness was depicted for malaria episodes in which any non-falciparum species alone or in combination with any other *Plasmodium* species was detected by qPCR in the day 0 DBS sample. To assess efficacy, we determined crude cure rates for all non-falciparum species. We defined the day 28 cure rate on the basis of the post-hoc qPCR analysis, separately for each *Plasmodium* species other than *P falciparum* (*P malariae, P ovale curtisi, P ovale wallikeri*, and *P vivax)*. A patient was considered cured for the species of interest if the species was detected in the day 0 DBS and not detected in the day 28 DBS, irrespective of any other species being present in the day 28 DBS. Patients were considered not cured if the same species of interest was detected in a DBS taken on day 0, as well as in DBSs taken between day 4 and day 28.

When a mixed infection was present at day 0, the patient was considered for inclusion in the analysis of each species that was present in the day 0 sample, meaning that a patient could be allocated multiple times. *P falciparum* cure rates were not determined because no further qPCR analyses of pure *P falciparum* samples at day 28 were done.

Each cure rate is presented as the number and percentage of patients with exact Clopper Pearson 95% CIs overall, by species, and separately for each site. An additional summary of cure rates for the post-hoc per-protocol set is also provided.

### Role of the funding source

The funder was involved in the conceptualisation, funding acquisition, methods, project administration, resources, supervision, validation, and review and editing of the manuscript.

## Results

We included day 0 samples of 1502 malaria episodes in 1413 patients in this analysis; 750 malaria episodes in 715 patients from the DR Congo site and 752 malaria episodes in 698 patients from the Gabon site. Of these, 64 (9·0%) patients at the DR Congo site and 96 (13·8%) at the Gabon site (160 [11·3%] overall) had multiple episodes of malaria. On day 28, three samples were qPCR-positive for non-falciparum malaria at the DR Congo site and none at the Gabon site. There were no non-falciparum-positive samples from an unscheduled visit between days 0 and 28 on either site.

Overall, slightly more randomly assigned malaria events occurred in male participants than in female participants (table 1), which was similar between the two sites (405 [54**·**0%] events in male participants and 345 [46**·**0%] in female participants in DR Congo, and 399 [53**·**1%] in male participants and 353 [46**·**9%] in female participants in Gabon). The overall median age in this randomly selected population was 10·0 years (SD 14·8) and was also similar at the DR Congo and Gabon sites (9·0 years [13·9] at the DR Congo site and 11·0 years [15·6] at the Gabon site). The majority of infections overall and at each of the two sites occurred in participants aged 5–12 years (634 [42·2%] overall, 314 [41·9%] at the DR Congo site, and 320 [42·6%] at the Gabon site). The mean body-mass index was similar overall and at each of the two sites at the DR Congo and Gabon sites (17·4 kg/m^2^, SD 4·5, overall; 16·8 kg/m^2^, 4·1, at the DR Congo site; and 18·0 kg/m^2^, 4·7, at the Gabon site).

**Table 1:** Demographic characteristics overall and by day 0 species

		**All malaria episodes (n=1502)**	Plasmodium falciparum **mono-infections (n=1237)**	Plasmodium malariae **mono-infections (n=6)**	Plasmodium ovale curtisi **mono-infections (n=2)**	Plasmodium ovale wallikeri **mono-infections (n=1)**	**Mixed infections (n=183)**	No infection*(n=73)
Sex								
	Male	804 (53·5%)	656 (53·0%)	4 (66·7%)	1 (50·0%)	0	107 (58·5%)	36 (49·3%)
	Female	698 (46·5%)	581 (47·0%)	2 (33·3%)	1 (50·0%)	1 (100%)	76 (41·5%)	37 (50·7%)
Age, years		10·0 (5·0–18·0)	10·0 (5·0–19·0)	14·5 (5·0–24·0)	38·5 (15·0–62·0)	11·0 (11·0–11·0)	9·0 (6·0–14·0)	8·0 (5·0–15·0)
Age category, years								
	<5	298 (19·8%)	250 (20·2%)	1 (16·7%)	0	0	29 (15·8%)	18 (24·7%)
	5–12	634 (42·2%)	494 (39·9%)	2 (33·3%)	0	1 (100%)	103 (56·3%)	34 (46·6%)
	>12 to <18	179 (11·9%)	151 (12·2%)	0	1 (50·0%)	0	23 (12·6%)	4 (5·5%)
	≥18	391 (26·0%)	342 (27·6%)	3 (50·0%)	1 (50·0%)	0	28 (15·3%)	17 (23·3%)
BMI, kg/m^2^		17·4 (4·5)	17·6 (4·6)	18·1 (4·1)	20·2 (3·8)	26·0	16·5 (3·2)	17·2 (5·0)

Data are n (%), median (IQR), or mean (SD) for the post-hoc intention-to-treat analysis set. Missing values were not included in the calculation of percentages. There were no patients infected with *Plasmodium vivax.* BMI=body-mass index.

* Assessed by quantitative PCR.

Of the 1502 malaria episodes on day 0, 1237 [82·4%] were *P falciparum* mono-infections, six (0·4%) were *P malariae* mono-infections, three (0·2%) were *P ovale* spp mono-infections (two [0·1%] *P ovale curtisi* and one [0·1%] *P ovale wallikeri*), 183 (12·2%) were mixed *Plasmodium* spp infections, and 73 (4·9%) were negative in qPCR. No patient was reported with a *P vivax* infection.

182 (12·1%) episodes in the post-hoc subset were mixed *P falciparum* infections, with one mixed infection not including this species (*P malariae* plus *P ovale curtisi*; table 2). Most *P malariae* (71 [95·9%]) and *P ovale* spp (122 [95·3%]) infections occurred as part of mixed infections. Mixed Plasmodium infections were equally balanced between the site in DR Congo (97 [13·1%]) and the site in Gabon (86 [12·5%]; table 2). The overall mean parasite counts per μL on day 0, as determined by qPCR analysis, were 13115 per μL (SD 36645) for *P falciparum*, 283 per μL (650) for *P malariae*, 272 per μL (712) for *P ovale curtisi*, and 1808 per μL (8536) for *P ovale wallikeri* (table 3).

**Table 2:** Parasite species on day 0 determined by quantitative PCR by centre and overall

	**Democratic Republic of the Congo (n=750)**	**Gabon (n=752)**	**Total (n=1502)**
**Number of episodes with an infection**			
*Plasmodium falciparum*	717 (95·6%)	702 (93·4%)	1419 (94·5%)
*Plasmodium malariae*	69 (9·2%)	59 (7·8%)	128 (8·5%)
*Plasmodium ovale curtisi*	20 (2·7%)	27 (3·6%)	47 (3·1%)
*Plasmodium ovale wallikeri*	16 (2·1%)	11 (1·5%)	27 (1·8%)
*Plasmodium vivax*	0	0	0
**Number of episodes with mono-infection**			
*P falciparum*	621 (82·8%)	616 (81·9%)	1237 (82·4%)
*P malariae*	1 (0·1%)	5 (0·7%)	6 (0·4%)
*P ovale curtisi*	0	2 (0·3%)	2 (0·1%)
*P ovale wallikeri*	0	1 (0·1%)	1 (0·1%)
*P vivax*	0	0	0
No parasites found	31 (4·1%)	42 (5·6%)	73 (4·9%)
**Number of episodes with mixed infections**			
*P falciparum* plus *P malariae*	61 (8·1%)	51 (6·8%)	112 (7·5%)
*P falciparum* plus *P ovale* spp	29 (3·9%)	32 (4·3%)	61 (4·1%)
*P falciparum* plus *P malariae* plus *P ovale* spp	6 (0·8%)	3 (0·4%)	9 (0·6%)
*P malariae* plus *P ovale curtisi*	1 (0·1%)	0	1 (0·1%)

Data are n (%) for the post-hoc intention-to-treat analysis set. *P ovale* spp values below the limit of quantification (<5 per μL) were imputed as 4·9 per μL.

**Table 3:** Species-specific parasitaemia on day 0 determined by qPCR by centre and overall

	**Democratic Republic of the Congo**	**Gabon**	**Total**
Plasmodium falciparum**, per μL**			
Available observations	717	702	1419
Mean	16 517·7 (406 934·1)	9638·9 (316 320·6)	136 114·7 (366 645·1)
Range	1·0–4 463 661·1	1·0–3 016 221·7	1·0–4 436 661·1
Plasmodium malariae**, per μL**			
Available observations	69	59	128
Mean	394·0 (840·8)	154·1 (253·6)	283·4 (649·8)
Range	6·1–5250·0	5·2–1370·0	5·2–5250·0
Plasmodium ovale curtisi**, per μL**			
Available observations	20	27	47
Mean	211·1 (501·4)	316·8 (841·9)	271·8 (712·2)
Range	14·7–2280·0	6·9–4280·0	12·2–4280·0
P ovale walliker**i, per μL**			
Available observations	16	11	27
Mean	2908·5 (116 093·1)	207·0 (285·7)	1807·9 (8535·5)
Range	4·9–446 500·0	4·9–1000·0	4·9–44 500·0
Plasmodium vivax**, per μL**			
Available observations	0	0	0
Mean	NA	NA	NA
Range	NA	NA	NA

Data are n, mean (SD), or range for the post-hoc intention-to-treat analysis set. *P ovale* spp values below the limit of quantification (<5 per μL) were imputed as 4·9 per μL. NA=not applicable.

Patients with a *P falciparum, P malariae, P ovale curtisi*, or *P ovale wallikeri* mono-infection all had headache and rigours or chills as the two most commonly reported malaria symptoms on day 0. By contrast with patients with a *P falciparum* mono-infection, patients with a *P malariae, P ovale curtisi*, or *P ovale wallikeri* mono-infection did not show any events of sweating, jaundice, and haepatomegaly. Patients with mixed *Plasmodium* infections commonly reported symptoms of headache (124 [67·8%] of 183 episodes), rigours or chills (92 [50·3%]), cough (67 [36·6%]), and loss of appetite or anorexia (61 [33·3%]). Mean body temperatures at day 0 were similar overall and for patients infected with a *P falciparum* mono-infection, *P malariae* mono-infection, *P ovale curtisi* mono-infection*, P ovale wallikeri* mono-infection, or mixed-*Plasmodium* infection, ranging from 36·7°C to 37·2°C (SD 0·35 to 0·98). Detailed listings of signs and symptoms are shown in the appendix.

Overall, two samples on day 28 (both from DR Congo) had the same non-falciparum infection by qPCR as on day 0 and were further assessed by microscopy. In the microscopic reassessment, one slide was positive for *P falciparum* (qPCR result was *P falciparum* 9591 per μL, *P malariae* 6 per μL, *P ovale wallikeri* <5 per μL, with reappearance of *P malariae*) and the other slide was negative (*P ovale wallikeri* 6 per μL, with reappearance of *P ovale wallikeri*).

The cure rate at day 28 was defined on the basis of the post-hoc qPCR analysis separately for each *Plasmodium* species other than *P falciparum*. The day 28 cure rates in the post-hoc intention-to-treat analysis set for *P malariae, P ovale curtisi*, and *P ovale wallikeri* were 96**·**3% or higher (95% CIs from 81·0–99·9 to 95·7–100). We observed slightly higher point estimates of cure rates in patients with *P malariae* infection than in those infected with *P ovale curtisi* or *P ovale wallikeri*, but the 95% CIs crossed in all subanalyses (table 4). In the post-hoc per-protocol set, we observed similar cure rates at day 28, with a likewise overall cure rate of 96·3% or higher (95% CIs from 81·0–99·9 to 95·7–100; table 5).

**Table 4:** Cure rate at day 28 by species, by centre and overall

	**Democratic Republic of the Congo**	**Gabon**	**Total**
Plasmodium malariae			
Available observations	69	59	128
Number of patients with cure on day 28	68 (98·6%; 92·2–100)	59 (100·0%; 93·9–100)	127 (99·2%; 95·7–100)
Plasmodium ovale curtisi			
Available observations	20	27	47
Number of patients with cure on day 28	20 (100·0%; 83·2–100)	26* (96·3%; 81·0–99·9)	46 (97·9%; 88·7–99·9)
P ovale wallikeri			
Available observations	16	11	27
Number of patients with cure on day 28	15 (93·8%; 69·8–99·8)	11 (100%; 71·5–100)	26 (96·3%; 81·0–99·9)

Data are n or n (%; 95% Clopper Pearson CI) for the post-hoc intention-to-treat analysis set. Percentages are based on the number of available observations. No *Plasmodium vivax* infections were detected.

* One non-falciparum negative day 28 sample was collected with relevant delay (on day 99), and was thus conservatively counted as failure in the post-hoc intention-to-treat analysis set.

**Table 5:** Cure rate at day 28 by species, by centre and overall

	**Democratic Republic of the Congo**	**Gabon**	**Total**
Plasmodium malariae			
Available observations	68	59	127
Number of patients with cure on day 28	67 (98·5%; 92·1–100)	59 (100%; 93·9–100)	126 (99·2%; 95·7–100)
Plasmodium ovale curtisi			
Available observations	20	26	46
Number of patients with cure on day 28	20 (100%; 83·2–100)	26 (100%; 86·8–100)	46 (100%; 92·3–100)
P ovale wallikeri			
Available observations	16	11	27
Number of patients with cure on day 28	15 (93·8%; 69·8–99·8)	11 (100%; 71·5–100)	26 (96·3%; 81·0–99·9)

Data are n or n (%; 95% Clopper Pearson CI) for the post-hoc per-protocol analysis set. Percentages are based on the number of available observations. No *Plasmodium vivax* infections were detected.

## Discussion

With this post-hoc analysis of the CANTAM-Pyramax real-world, safety, tolerability, and efficacy trial, we aimed to provide high-quality data to fill the current gap of high-grade evidence for treatment recommendations for blood-stage clearance of non-falciparum and non-vivax malaria.^2^, ^3^ The results of this study provide important evidence supporting the use of pyronaridine–artesunate for the treatment of blood stages of mixed *Plasmodium* malaria and malaria caused by mono-infections of *P malariae, P ovale curtisi*, and *P ovale wallikeri* in a real-world setting. To our knowledge, to date, this is the largest cohort in a study systematically assessing the treatment outcome of patients with such infections.

The distributions of baseline characteristics in this post-hoc subset were similar between both study sites, which suggests an unbiased randomisation process for the selection of samples. The underlying expectation that approximately 10% of the 1502 randomly selected malaria episodes would be non-falciparum mono-infections or mixed *Plasmodium* infections was exceeded by the observed 192 (12·8%) such infections in this analysis, which were equally distributed between the urban site in DR Congo and the rural site in Gabon. 183 (12·2%) of all episodes were mixed infections. This underlines the epidemiological importance of non-falciparum and mixed *Plasmodium* malaria in central Africa and the associated need for antimalarials with confirmed effectiveness for the treatment of these neglected *Plasmodium* infections.

Determining antimalarial treatment efficacy for non-falciparum and mixed *Plasmodium* infections can be challenging. Information about the epidemiology of *P malariae* and *P ovale* spp is scarce, which can make it difficult to identify suitable geographical settings where sufficiently large sample sizes can be obtained for clinical trials in this field. Additionally, the diagnosis of non-falciparum malaria requires microscopists to be well trained in identifying potential study participants. In case of reappearance of parasites during the observational period, no established methods are available to reliably distinguish recrudescence from reinfection and relapse, if applicable, which might lead to overestimation or underestimation of failure rates. In this post-hoc analysis, we chose the more conservative approach to consider reappearance of the same *Plasmodium* species within the follow-up period as failure.

To reflect a real-world setting, patients with malaria were recruited in the CANTAM-Pyramax trial on the basis of either a positive RDT result (using only tests with WHO prequalification detecting HRP-2 antigens alone or HRP-2 and pLDH or aldolase antigens) or malaria microscopy—depending on what was routine procedure at the respective sites. On the basis of the fact that some RDTs were targeting *P falciparum* infection alone and that microscopic identification of low-level parasitaemia of non-falciparum malaria is challenging, the observed prevalence in this study might underestimate somewhat the true prevalence of non-falciparum malaria in this setting.

Fewer than 5% of samples of patients recruited on the basis of RDT tests had a negative result in qPCR testing. This discrepancy of results might be explained by the test characteristics of RDTs. Positive predictive values (PPVs) and negative predictive values of RDTs for clinical malaria have been modelled previously,^21^ and PPVs differed considerably between countries when used for screening,^21^ suggesting that there are substantial error rates in RDT-based test-and-treat algorithms in low-PPV settings (ie, ≤10% of false positives). Positive RDT test results might, for example, be explained by successfully treated malaria episodes within up to 4–6 weeks before RDT testing due to persistence of circulating antigens. This suggests that the presence of a positive RDT result for a proportion of patients, particularly in low-PPV settings, might not always reflect presence of infection at the time of testing.^21^, ^22^ This finding in this subset is thus in line with the test characteristics of RDTs. Additionally, we cannot rule out that degradation of DNA due to handling, storage, and shipment of samples might also have played a role in subsequent testing of individuals with low-level parasitaemia. In summary, the low proportion of discrepant test results using different diagnostic assays seems in line with the performance characteristics of the tests.

The microscopic reassessment of qPCR-positive day 28 samples showed one slide being microscopically positive for *P falciparum* and one microscopically negative slide, suggesting there were submicroscopic non-falciparum parasitaemia in both samples. The effect of antimalarial treatment regimens with and without 8-aminoquinolines on *P falciparum* gametocytes have been described previously, and a 2022 publication has detailed the specific clinical effects of pyronaridine–artesunate on the sexual stages of this species.^23^ However, the effects of antimalarials on the sexual stages of *Plasmodium malariae* and *Plasmodium ovale* spp, and the characteristics of those stages, have not been described conclusively so far.^12^, ^24^ Therefore, we cannot rule out that these submicroscopic parasitaemias were due to sexual stages of said non-falciparum parasite species that, to date, cannot be reliably distinguished from asexual stages with the available molecular methods.

Overall cure rates on day 28 in this post-hoc analysis were very good for all non-falciparum species and similar to the effectiveness of pyronaridine–artesunate in *P falciparum* and *P vivax* (>95%).^12^, ^25^, ^26^, ^27^ Overall cure rates were similar in mixed infections. The only outlier was the noticeably decreased cure rate (93·8%) for *P ovale wallikeri* at the DR Congo site. However, this is based on one observation alone, which had such a big effect because of the low sample size in this group (one of 16 observations). Given that the clinical trial was an effectiveness trial with unsupervised treatment intake on days 2 and 3, these high cure rates further support the use of pyronaridine–artesunate in clinical routine. Therefore, our results give confidence that pyronaridine–artesunate can be used to effectively treat blood-stage infections of all African *Plasmodium* spp reported in this study. This finding can simplify the future management of patients with malaria, given the challenges of routine diagnostics of non-falciparum and mixed-species malaria by RDT and microscopy.

In this sample subset, *P malariae* was more prevalent than *P ovale* spp, which reflects what is reported from other endemic regions.^28^ We observed more *P ovale curtisi* than *P ovale wallikeri* episodes; this differs from data from returning travellers, but it is in line with previous prospective research done in Gabon.^14^, ^29^ One reason for this might be the different exposure profiles (eg, long-term resident in an endemic region *vs* short-time traveller from an endemic region diagnosed in a non-endemic region).^30^ To date, data on *Plasmodium knowlesi* have only been reported from southeast Asia.

For a long time, *P vivax* infection was thought to be limited to individuals expressing the Duffy antigen–chemokine receptor gene, which served as explanation for the absence of vivax infections in large parts of Africa. However, over the past years, more and more confirmed vivax cases were reported not only from the African continent, but also at the same time from Duffy-negative individuals.^31^ This seems to be attributable to increased structural polymorphisms in erythrocyte-invading genes in parasite isolates from this geographical area.^32^ PCR-confirmed *P vivax* infections have also been previously reported in DR Congo.^33^ However, this finding is not reflected in our data.

A key limitation of this post-hoc analysis is that we observed only nine non-falciparum mono-infections in this large subset of malaria episodes. For this analysis, outcomes for each *Plasmodium* species were determined no matter whether they occurred as part of a mono-infection or mixed infection. We are not aware of any data showing reduced effectiveness of antimalarials in mono-infections compared with mixed *Plasmodium* infections, and thus we are confident that these data provide reliable estimates for both mono-infections and mixed-species infections with these *Plasmodium* species. Additionally, it might not be ruled out that some non-falciparum infections were below the threshold of detection at initial assessment due to quantitative suppression by *P falciparum* or due to primer competition. However, as this study aimed to assess the therapeutic effectiveness of pyronaridine–artesunate, the analysis was restricted to only those patients with a positive PCR at recruitment.

In summary, this post-hoc analysis provides high-quality evidence for the high efficacy and clinical usefulness of pyronaridine–artesunate combination therapy for the treatment of *P ovale, P malariae*, and mixed-species malaria.

## Data sharing

All relevant data are presented in this Article and the appendix. The data underlying the results presented in the study are available from Medicines for Malaria Venture (https://www.mmv.org) on reasonable request.

## Declaration of interests

IB-F and SD are full-time employees of Medicines for Malaria Venture (MMV). JS is a full-time employee of Shin-Poong Pharmaceutical. RM and SA-B are consultants paid by Shin-Poong Pharmaceutical. All other authors declare no competing interests.
