# Supplementary material for: Effectiveness of pyronaridine-artesunate against Plasmodium malariae, Plasmodium ovale spp, and mixed-Plasmodium infections: a post-hoc analysis of the CANTAM-Pyramax trial
Source: Lancet Microbe. 2022 Aug;3(8):e598–605. doi: 10.1016/S2666-5247(22)00092-1 (PMC9329129; doi:10.1016/S2666-5247(22)00092-1)
Supplement: Supplementary appendix [file mmc1.pdf]

THE LANCET
Microbe

Supplementary appendix

This appendix formed part of the original submission and has been peer reviewed. We post it as supplied by the authors.

Supplement to: Groger M, Tona Lutete G, Mombo-Ngoma G, et al. Effectiveness of pyronaridine-artesunate against *Plasmodium malariae*, *Plasmodium ovale* spp, and mixed-*Plasmodium* infections: a post-hoc analysis of the CANTAM-Pyramax trial. *Lancet Microbe* 2022; published online May 30. https://doi.org/10.1016/S2666-5247(22)00092-1.

Supplementary Table 1 Signs and Symptoms of Malaria at Baseline by Centre and Overall (Post-hoc Analysis Set)

	**All participants N = 1502 n (%)**	***P. falciparum* monoinfections N = 1237 n (%)**	***P. malariae* monoinfections N = 6 n (%)**	***P. ovale* spp. monoinfections N = 3 n (%)**	***P. ovale curtisi* monoinfections N = 2 n (%)**	***P. ovale wallikeri* monoinfections N = 1 n (%)**	**Mixed infections N = 183 n (%)**	**No infection (by qPCR) N = 73 n (%)**
Rigours/ Chills	779 (51·9)	657 (53·1)	2 (33·3)	2 (66·7)	1 (50·0)	1 (100·0)	92 (50·3)	26 (35·6)
Sweating	420 (28·0)	352 (28·5)	0	0	0	0	46 (25·1)	22 (30·1)
Headache	1017 (67·7)	849 (68·6)	2 (33·3)	2 (66·7)	2 (100·0)	0	124 (67·8)	40 (54·8)
Nausea	307 (20·4)	267 (21·6)	0	2 (66·7)	1 (50·0)	1 (100·0)	34 (18·6)	4 (5·5)
Vomiting	401 (26·7)	356 (28·8)	0	1 (33·3)	1 (50·0)	0	37 (20·2)	7 (9·6)
Cough	566 (37·7)	468 (37·8)	2 (33·3)	1 (33·3)	0		67 (36·6)	28 (38·4)
Loss of appetite/ Anorexia	694 (46·2)	611 (49·4)	1 (16·7)	2 (66·7)	2 (100·0)	0	61 (33·3)	19 (26·0)
Fatigue	870 (57·9)	754 (61·0)	3 (50·0)	2 (66·7)	1 (50·0)	1 (100·0)	84 (45·9)	27 (37·0)
Myalgia	435 (29·0)	381 (30·8)	1 (16·7)	2 (66·7)	1 (50·0)	1 (100·0)	39 (21·3)	12 (16·4)
Jaundice	11 (0·7)	11 (0·9)	0	0	0	0	0	0
Hepatomegaly	8 (0·5)	6 (0·5)	0	0	0	0	1 (0·5)	1 (1·4)
Splenomegaly	138 (9·2)	104 (8·4)	0	1 (33·3)	1 (50·0)	0	29 (15·8)	4 (5·5)
Other	431 (28·7)	364 (29·4)	2 (33·3)	2 (66·7)	1 (50·0)	1 (100·0)	45 (24·6)	18 (24·7)

Abbreviations: P. = Plasmodium; N = total number of malaria episodes; n = number of observations;
